# Case Report: Synergistic central nervous system depression of baclofen and pregabalin: clinical pharmacist-driven case analysis and case review

**DOI:** 10.3389/fphar.2025.1598971

**Published:** 2025-06-05

**Authors:** Yitong Xie, Danwei Wu, Peihao Jin, Lu Mao, Jiancun Zhen, Wei Zhang

**Affiliations:** ^1^ School of Pharmaceutical Sciences, Capital Medical University, Beijing, China; ^2^ Department of Pharmacy, Beijing Jishuitan Hospital, Capital Medical University, Beijing, China; ^3^ Department of Spine Surgery, Beijing Jishuitan Hospital, Capital Medical University, Beijing, China

**Keywords:** baclofen, pregabalin, drug-induced CNS depression, renal impairment, synergistic toxicity

## Abstract

**Background:**

Baclofen, a γ-aminobutyric acid (GABA) derivative, and Pregabalin, a GABA analogue, are widely prescribed for muscle spasm and neuropathic pain. This first-reported case demonstrates synergistic central nervous system (CNS) depression of Baclofen (30 mg/day) and Pregabalin (300 mg/day) in a patient with mild renal impairment [estimated glomerular filtration rate, (eGFR) = 77.26 mL/min].

**Case Presentation:**

A 68-year-old female with renal impairment developed progressive CNS depression (somnolence, coma) following combined Baclofen (30 mg/day) and Pregabalin (300 mg/day) therapy after spinal fusion surgery. CNS depression was completely resolved 48 h after drug discontinuation.

**Conclusion:**

Clinicians should exercise heightened caution when combining Baclofen and Pregabalin in renal impairment. Dose adjustments based on creatinine clearance (CLcr) are strongly recommended. Particular attention should be given to initiating therapy with reduced starting doses in patients at elevated risk of CNS depression.

## Introduction

Baclofen, a GABA derivative, exerts antispasmodic effects by agonizing postsynaptic GABA-β receptors in spinal and supraspinal regions, thereby amplifying inhibitory neurotransmission. It is clinically utilized to alleviate skeletal muscle spasms associated with diverse neurological disorders. Initial therapy should employ a low-dose regimen (typically 5 mg three times daily). This graduated dosing strategy reduces the risk of dose-dependent CNS depression by allowing gradual adaptation of GABAergic neurotransmission systems (Fromm, 1994). Common CNS adverse reactions included dizziness (spinal 1.7%, cerebral 2.4%), headache (spinal 1.6%, cerebral 6.6%), and confusion (spinal 0.5%, cerebral 0.5%). Severe events such as generalized seizures (0.5%) and death (0.2% in spinal cohort) were rarely reported. Notably, Baclofen may paradoxically exacerbate muscle spasms in susceptible individuals and lower seizure thresholds, particularly in epilepsy patients. Cerebral-origin cases showed higher rates of nausea/vomiting (6.6%), speech disorders (0.5%), and respiratory depression (1.4%). Pharmacokinetically, it demonstrates rapid absorption with peak plasma concentrations achieved within 0.5–1.5 h post-dose, followed by predominant renal excretion of the unchanged compound (approximately 85%). The drug displays linear pharmacokinetics (dose-proportional AUC) with a volume of distribution (Vd) of 0.7 L/kg and approximately 30% plasma protein binding across therapeutic concentrations (10–300 ng/mL). The plasma elimination half-life (T_1/2)_ averages 3–4 h in normal renal function but is significantly prolonged in renal impairment. This accumulation in renal impairment can enhance systemic exposure and CNS penetration (Sellersville, 2016; Kent et al., 2020).

Pregabalin, a GABA analogue, is primarily prescribed for managing various types of neuralgia, including postherpetic neuralgia, diabetic peripheral neuralgia, fibromyalgia, partial seizures, and spinal cord injury-associated pain. It acts as a presynaptic calcium channel α2-δ subunit inhibitor, reduces excitatory neurotransmitter release *via* voltage-dependent calcium current suppression, synergizing with postsynaptic GABAergic inhibition (Alles et al., 2020; Pin and Prézeau, 2007). It exhibits rapid absorption with peak plasma concentrations attained within 1 h post-dose, near-complete oral bioavailability (≥90%) with linear pharmacokinetics (steady-state achieved within 24–48 h). The drug demonstrates a low Vd (0.56 L/kg) and negligible plasma protein binding. Adverse reactions to Pregabalin commonly manifest early in treatment and exhibit dose dependency; initiating therapy at lower doses with gradual titration substantially mitigates their occurrence. In controlled studies, dizziness and somnolence emerged as the most common adverse reactions leading to discontinuation (4% incidence for each), attributable to amplified inhibition of glutamatergic transmission in the locus coeruleus and hypothalamus (Al-Husseini et al., 2018). The recommended evening-first dosing strategy minimizes daytime functional impairment by synchronizing peak sedative effects (e.g., somnolence, dizziness) with nocturnal sleep periods—a particularly beneficial approach for patients with comorbid insomnia or anxiety disorders. This temporal alignment not only improves medication adherence but also addresses circadian-triggered neuropathic pain exacerbations. Renal clearance directly proportional to CLcr (For the average adult, eGFR can be used as an alternative to CLcr to adjust drug dosage. Estimated from serum creatinine (mg/dL) determination using the Cockcroft and Gault equation) in governs elimination, with a mean T_1/2_ of 6.3 h in normal renal function. In mild renal impairment (CLCr 30–60 mL/min), prolonged T_1/2_ elevates plasma exposure and CNS accumulation. Pharmacokinetic parameters exhibit minimal inter-individual variability (<20%), predictable from single-dose data (Derry et al., 2019; Bockbrader et al., 2010; FDA, 2025).

Although Baclofen and Pregabalin lack pharmacokinetic interactions, both demonstrate pharmacodynamic synergism with other CNS depressants (Riaz et al., 2020; Jeyaselvasenthilkumar et al., 2019). Notably, their complementary mechanisms—with Baclofen modulating spinal GABA-β activity and Pregabalin acting on calcium channels—may potentiate neural inhibition through convergent pathways. This synergistic risk manifests not only in renal impairment but also with standard initial doses administered without reduction, mandating particular vigilance in polypharmacy scenarios.

This report details a patient with renal impairment who developed somnolence, coma, and unconsciousness following combined Baclofen and Pregabalin administration at higher initial therapeutic doses use after spinal surgery. This case highlights the CNS risks associated with this drug combination in patient’s with renal impairment. Through case analysis and literature review, we discussd the pharmacodynamically synergistic mechanisms involving GABA-β receptors modulation and calcium channel inhibition, clinical presentations, and management strategies for CNS depression, providing recommendations for cautious dosing adjustments.

### Case description

A 68-year-old woman (height 154 cm, weight 65 kg) was admitted to the hospital with lumbar spinal stenosis, suffering from hypertension, coronary artery disease, gastroesophageal reflux disease, hyperuricemia, renal impairment (eGFR = 77.26 mL/min) and was regularly taking Amlodipine Besylate 5 mg qd; Metoprolol Tartrate 25 mg qd; Isosorbide Dinitrate 5 mg bid; Atorvastatin 20 mg bid; Aspirin 100 mg qd (which had been stopped before surgery); Omeprazole 10 mg bid; Allopurinol 100 mg bid. The patient’s medication allergy history was limited to palpitations following intravenous levofloxacin and etimicin administration during a previous hospitalization, with no other documented drug allergies or adverse drug reactions.

On 21 December 2023, the patient underwent spinal fusion *via* an obliquely lateral approach including discectomy, interbody fusion implantation, implant fusion, and posterior pedicle screw internal fixation. Intraoperative blood loss was 800 mL, and the operation took 8 h and 45 min. The operation went smoothly, and the patient returned to the ward.

**FIGURE 1:**
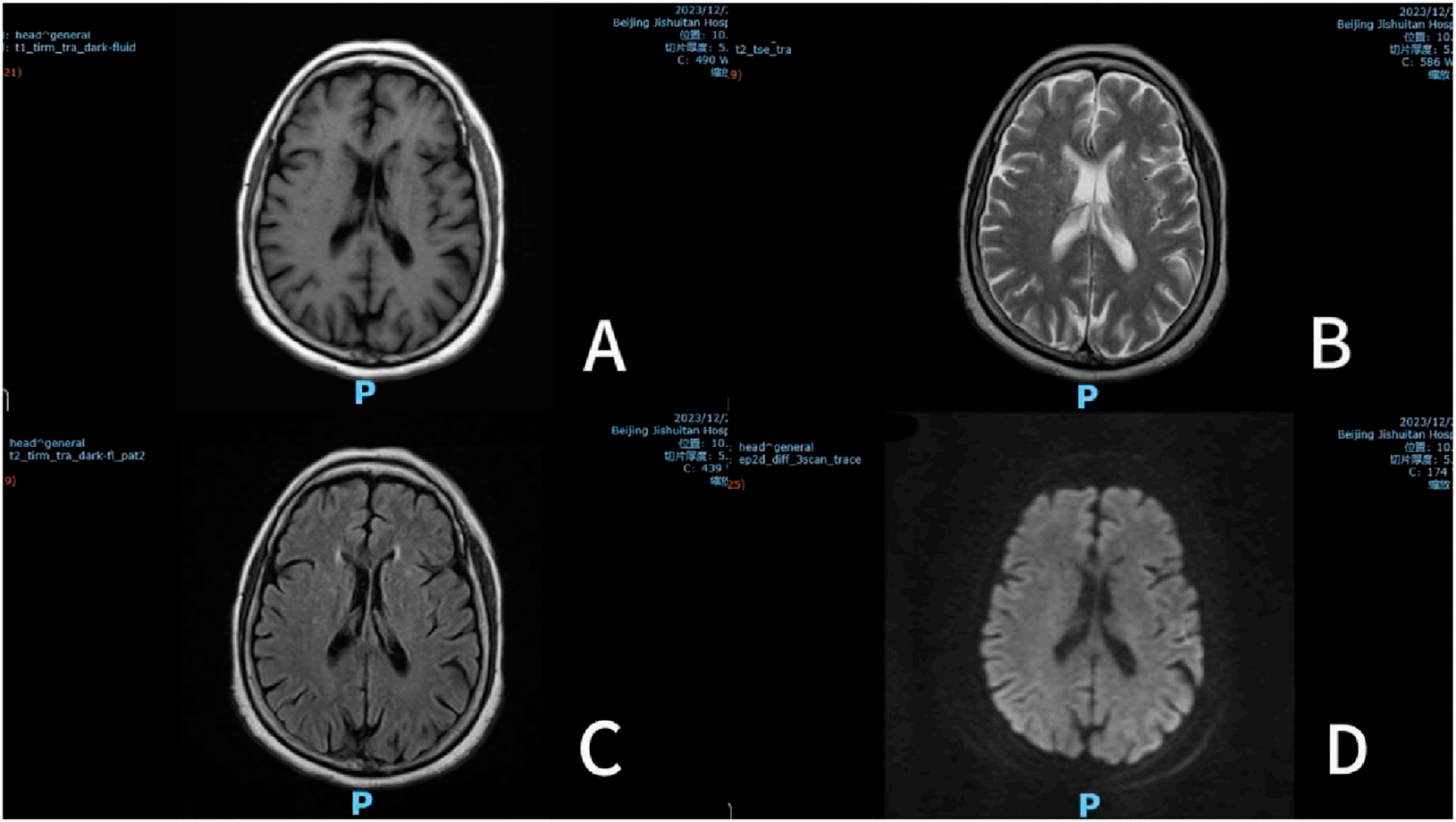
Multisequence brain MRI (December 27, 2023). Note: **(A)** T1 weighted imaging (T1WI); **(B)** T2 weighted image (T2WI); **(C)** Fluid attenuated inversion recovery (T2 FLAIR); **(D)** Diffusion-weighted imaging (DWI).

On December 26, the patient complained of right lower limb pain and was prescribed Baclofen 10 mg tid combined with Pregabalin 150 mg bid. At 2:00 p.m., she took the first dose (Pregabalin 150 mg and Baclofen 10 mg), followed by a second dose at 8:00 p.m. Somnolence developed at 10:00 p.m.that evening. By 7:30 a.m. on December 27, the patient became unconscious and regained consciousness at 10:00 a.m. Subsequently, Magnetic Resonance Imaging (MRI), (Figure 1) was performed and a neurology consultation was requested. The brain MRI revealed an old lacunar infarct and a demyelinating focus in the cerebral white matter. The neurology team conducted a comprehensive evaluation, including neurological physical examination and imaging review, which confirmed the absence of acute disease progression. At 11:00 a.m. on December 27, the patient received the third dose. Her condition deteriorated, progressing to coma by 2:00 p.m., accompanied by fever (peak temperature 38.5°C). The fever resolved after administration of lysine acetylsalicylate 0.9 g and did not recur. The biochemical indexes were tested for the first time on December 27 after the operation, among which Ca 2.12 mmol/L, IP 0.7 mmol/L, K 3.70 mmol/L, Na 137 mmol/L, Cl 106 mmol/L,CRP 55.08 mg/L. Prior to the formal pharmaceutical consultation initiated by the clinical team to evaluate the adverse event, the clinical pharmacist proactively reviewed the medical records to understand the patient’s clinical status. Then, the clinical pharmacist proactively conducted a interview with the patient’s family, systematically documenting medication history (including OTC/herbal use), allergy profiles, prior CNS adverse reactions, and temporal details of symptom onset/progression. This assessment demonstrated a clear temporal relationship between CNS depression and the combined administration of Baclofen and Pregabalin, with no prior similar reactions documented. Following exclusion of primary neurological disease progression through neurology consultation, the Naranjo Scale assessment yielded a score of 8 (probable causality) (Naranjo et al., 1981). Based on comprehensive medication reconciliation and causality analysis, the pharmacist ultimately recommended immediate discontinuation of both medications, which was implemented. Neurological recovery occurred at 17:30 with complete symptom resolution, and no subsequent CNS depression was observed.

## Discussion

To our knowledge, most of the previous investigations only focused exclusively on single-agent toxicity. This represents the first documented case of synergistic CNS depression from combined Baclofen and Pregabalin administration at doubled initial doses in a renally impaired patient. Firstly, both Baclofen and Pregabalin have been reported to have CNS depression such as somnolence (Jeyaselvasenthilkumar et al., 2019; Mishaal et al., 2020; Ibeson et al., 2021; Pathak et al., 2019; Shah et al., 2020; Varma and Bajpai, 2022; de Marcellus et al., 2019; Khazneh et al., 2018; Wolf et al., 2018; Takia et al., 2021; Anand and Kaplan, 2018). Secondly, the patient developed somnolence 2 h post-second dose administration (December 26), aligning with peak plasma concentrations of both drugs (Baclofen T_max_ 0.5–1.5h; Pregabalin T_max_ ≤1 h). Subsequent coma onset at 24 h post-initiation (December 27) corresponded to near-steady-state levels (Baclofen T_1/2_ 3–4h; Pregabalin T_1/2_ 6.3 h requiring 24–48 h to achieve steady state). Complete symptom resolution followed drug discontinuation without recurrence. This temporal correlation strongly supports CNS depression. Thirdly, we systematically excluded alternative etiologies through pharmacological, neurological, and metabolic evaluations. The patient had no use of any opioids before, and although antihypertensive drugs like Amlodipine Besylate and Metoprolol Tartrate may cause dizziness and drowsiness, the patient has been taking these medications regularly without experiencing similar adverse reactions. Anesthetic agents administered on December 21 (sevoflurane, propofol, etomidate) exceeded their elimination half-lives (propofol T_1/2_ = 3–12 h) by 54 h before symptom onset. Neurology consultation confirmed no acute neurovascular progression through imaging and clinical assessment. Postoperative hypophosphatemia (0.7 mmol/L) and hypokalemia (3.70 mmol/L) resolved rapidly with supplementation, inconsistent with the severity or timeline of consciousness impairment. These evidentiary pillars yielded a Naranjo Score of 8. Although therapeutic drug monitoring (TDM) could theoretically strengthen causality arguments, current Chinese guidelines (Division of Therapeutic Drug Monitoring et al., 2022) did not recommend Baclofen/Pregabalin TDM due to absent defined toxic thresholds. Thus, Naranjo assessment remains the principal validation method here, providing a mechanistic analysis foundation.

CNS depression induced individually by Pregabalin and Baclofen is well-documented, including severe outcomes such as coma, respiratory suppression, and even fatal events. Compared to patients with normal renal function, those with renal impairment demonstrate a higher incidence of these adverse reactions, correlating with the severity of renal impairment. However, this case diverges from prior observations—our patient developed profound CNS depression despite only mild renal impairment (eGFR 77.23 mL/min), suggesting a previously unreported synergistic CNS depressive effect from their combined use. To systematically investigate the clinical manifestations, at-risk populations, and mechanistic basis of Baclofen- and Pregabalin-induced CNS depression—and to contextualize our findings—we conducted a literature review spanning studies from 2018 to 2024. Table 1 summarizes 16 peer-reviewed articles detailing adverse reactions to Baclofen and/or Pregabalin, providing critical evidence for comparative analysis. Notably, while our patient improved only with drug discontinuation, this underscores the need to expand therapeutic strategies and preventive measures for more severe cases.

**TABLE 1:** Cases of CNS adverse reactions (ADRs) caused by application of baclofen and/or Pregabalin

Author	Age/Gender	Kidney function	Reasons	Drug(s) involved	Time to first ADRs	Initial CNS symptom and others	Treatment	Outcome
Mishaal et al. (2020)	9/F	Acute Kidney Failure, RRT	Neuralgia	Baclofen 2.5 mg bid Pregabalin 25 mg qd	2 d	Consciousness Disorders, Dyspnea	Discontinuation, RRT	Cured after 25 d
	2.5/M	Renal insuffciency	Spasm	Baclofen 5 mg tid	4 d	Apnea, Bradycardia	Discontinuation, intravenous infusion therapy	Cured after 31 d
Riaz et al. (2020)	60/M	ESRD, RRT	Muscle pain	Baclofen 10 mg tid	Several hours	Delurium, Hallucinations	RRT	Cured after several days
Jeyaselvasenthilkumar et al. (2019)	64/M	CKD	Cervical myelopathy	Baclofen 10 mg bid	3 d	Coma, Apnea	RRT	Cured after 7 d
Ibeson et al. (2021)	21/F	Healthy	Muscle pain	Baclofen 5 mg tid	2 d	Somnolence, Coma, Encephalopathy	Mechanical ventilation	Cured after 3 d
Pathak et al. (2019)	58/F	ESRD	Muscle pain	Baclofen 10 mg bid carbamazepine 200 mg bid	1 d	Somnolence, Coma, Tremor	RRT	Better after 2 d
Al Marzouqi and Al Alawi (2023)	74/F	CKD	Muscle pain	Baclofen 10 mg bid	1 d	Consciousness Disorders	RRT	Better after 2 d
Gold et al. (2020)	35/M	Acute Kidney Failure	Spasm	Baclofen 7.5 mg qd	7 d	Encephalopathy	RRT	Better after 3 d
Shah et al. (2020)	6/M	Healthy	Misuse	1300 mg Baclofen (single dose)	Several hours	Coma, Encephalopathy	RRT	Better after 1 d
Varma and Bajpai (2022)	83/M	CKD-4	Hiccup	Baclofen 20 mg bid	7 h	Encephalopathy	RRT	Better after 2 d
	84/M	ESRD, RRT	Hiccup	Baclofen 10 mg bid	1 d	Somnolence, Encephalopathy	RRT	Cured after 3 d
	46/M	CKD-5	Hiccup	Baclofen 2.5 mg bid	4 d	Somnolence, Encephalopathy	RRT	Cured after 4 d
de Marcellus et al. (2019)	4/M	Healthy	Misuse	Baclofen overdose	Unknown	Coma	Mechanical ventilation	Cured after 3 d
Pisano et al. (2022)	43/M	HIV	Muscle pain	Baclofen 5 mg bid	2 d	Syncope	Discontinuation	Cured after 4 d
Khazneh et al. (2018)	47/F	ESRD, RRT	Muscle pain	Baclofen 25 mg	2 d	Coma, Delurium	RRT	Cured after 3 d
Wolf et al. (2018)	58/M	ERSD	Spasm	Baclofen 10 mg tid	Several days	Consciousness Disorders, Disorientation	RRT	Cured after 1 d
Takia et al. (2021)	8/F	Healthy	Misuse	Pregabalin 1125 mg (single dose)	1 h	Somnolence	RRT	Better after 4 h
Anand and Kaplan (2018)	41/F	CKD	Neuralgia	Unknown dose of Pregabalin	Unknown	Somnolence	Discontinuation, intravenous infusion therapy	Better after 3 d
Mousailidis et al. (2020)	36/F	Healthy	Anxiety	Pregabalin150 mg tid	3–4 days	Visual hallucinations, agitated	Gradual discontinuation	Cured after 5 d
Our case	68/F	Renal insuffciency	Muscle pain	Baclofen 10 mg tid Pregabalin150 mg bid	8h	Somnolence, Consciousness Disorders Coma	Discontinuation	Better after 1day

Note: CKD, chronic kidney disease; CKD-1 (eGFR>90 mL/min); CKD-2 (eGFR, 60–89 mL/min); CKD-3 (eGFR, 30–59 mL/min); CKD-4 (eGFR, 15–29 mL/min); CKD-5 (eGFR <15 mL/min); ESRD, end-stage renal disease; RRT, renal replacement therapy; HIV, human immunodeficiency virus.

Three prior studies employing the Naranjo Scale similarly identified a “probable” association between Baclofen and CNS depression, consistent with our findings (Mousailidis et al., 2020; Nahar et al., 2017; Dang et al., 2015). Mechanistically, Baclofen-induced somnolence and coma arise from GABA-β receptor-mediated hyperpolarization, where potassium efflux and reduced calcium influx suppress spinal reflexes and cortical arousal (Nahar et al., 2017). While gradual titration (initial dose 5 mg tid) remains standard, the clinical team opted for an accelerated regimen (10 mg tid) to rapidly alleviate the patient’s pain, prioritizing symptom control over theoretical titration protocols. This case underscores two pivotal determinants of CNS depression: supratherapeutic initial dosing and renal impairment. Firstly, the doubled initial Baclofen dose (10 mg vs. recommended 5 mg tid) directly precipitated rapid peak plasma concentrations (T_max_ 0.5–1.5 h) and cumulative drug exposure. Literature evidence from 12 renal-impaired cases (Riaz et al., 2020; Jeyaselvasenthilkumar et al., 2019; Mishaal et al., 2020; Ibeson et al., 2021; Pathak et al., 2019; Varma and Bajpai, 2022; Khazneh et al., 2018; Wolf et al., 2018; Al Marzouqi and Al Alawi, 2023; Gold et al., 2020) confirmed CNS depression occurrence even at therapeutic doses (5–30 mg/day Baclofen), highlighting the amplified risk from dose escalation. Secondly, renal impairment potentiated toxicity. Despite the patient’s mild renal impairment (eGFR 77.26 mL/min), age-related glomerulosclerosis and reduced renal blood flow significantly delayed drug clearance. Studies showed that even when CLcr was at the lower limit of normal (such as 60–90 mL/min), highly nephrotoxic drugs can still have a T_1/2_ that is 1.6–1.8 times longer (Mayer, 2009)^.^ Current guidelines recommend CLcr-based dose adjustments for Baclofen (Vlavonou et al., 2014).

Building on the dose-dependent risks of Pregabalin, recent studies demonstrate CNS toxicity even in patients with normal renal function. Mousailidis et al. (Mousailidis et al., 2020) reported a 36-year-old female developing visual hallucinations at 450 mg/day (threefold the minimum recommended initial dose), with symptom resolution upon discontinuation—a dosing pattern paralleling our patient’s accelerated regimen (150 mg bid vs the guideline-recommended 75 mg bid). Calandre et al. (Calandre et al., 2016) further quantified this dose-toxicity relationship, showing a direct correlation between Pregabalin plasma clearance and CLcr, with adverse reaction frequency and severity escalate proportionally to dose increments. Renal impairment exacerbates these risks through pharmacokinetic derangements. Although current guidelines enforce CLcr-based dose reductions (FDA, 2025), our findings reveal critical limitations in applying these thresholds to elderly populations, where age-related glomerular filtration decline amplifies drug accumulation even at nominally “mild” renal impairment (CLCr >60 mL/min). Such risks are missed by current guidelines.

In this case, a 68-year-old patient received combined Baclofen (10 mg tid) and Pregabalin (150 mg bid) for mixed pain components (muscle spasms and neuropathic pain) in the right lower limb following spinal fusion surgery. To achieve rapid analgesia, Baclofen and Pregabalin both begin with higher dose. Despite mild renal impairment (eGFR 77.23 mL/min), no explicit dose reduction was mandated per prescribing information for either drug in this renal function range, allowing therapeutic dosing for prompt pain control. In 2020, Mishaal et al. (Mishaal et al., 2020) reported a pediatric case where Baclofen (2.5 mg bid) and Pregabalin (25 mg qd) were combined for neuropathic pain in a patient requiring renal replacement therapy. CNS depression emerged 2 days post-initiation, temporally linked to Baclofen’s peak concentration (T_max_ 0.5–1.5 h). Authors attributed toxicity solely to Baclofen accumulation, dismissing Pregabalin’s contribution due to its administration 12 h prior to symptom onset. In contrast, our 68-year-old patient received higher initial doses with only mild renal impairment (eGFR 77.23 mL/min) and no RRT. Through multi-factorial analysis, we identified synergistic toxicity rather than single-drug accumulation. Mechanistically, Baclofen’s GABA-β agonism and Pregabalin’s α2-δ -mediated calcium channel inhibition amplify Ca^2+^ influx through distinct yet complementary pathways. This dual modulation depressed CNS arousal thresholds beyond monotherapy effects, particularly under renal impairment. Notably, both agents exhibit documented synergism with other CNS depressants (Sellersville, 2016; FDA, 2025) (as detailed in Table 2). Baclofen potentiates opioids *via* GABAergic pathways, while Pregabalin enhances benzodiazepine sedation through calcium channel modulation. Our findings extend these observations to their mutual interaction. Clinically, this case demonstrates that CLcr-based dose adjustments—though effective for single-agent regimens (FDA, 2025; Vlavonou et al., 2014)—fail to prevent toxicity in GABA-ergic polypharmacy. Systematic review data showing no CNS depression reports at CLcr 60–90 mL/min (Mayer, 2009) further indicate that the current guidelines overlook toxicity risks in patients with borderline renal function.

**TABLE 2:** Pharmacological interactions of baclofen and pregabalin.

Medicine	Therapeutic Class	Exemples	Mechanism	Clinical Manifestations
Baclofen	Opioids	Morphine, Fentanyl, Tramadol	Additive CNS depression	Severe respiratory depression
	Benzodiazepines	Diazepam, Midazolam	Synergistic CNS depression	Hypoxia, confusion, respiratory depression
	Antipsychotics	Quetiapine, Haloperidol	Additive CNS/QT effects	Respiratory depression, hypotension, arrhythmia
	Sedative-Hypnotics	Zolpidem, Zopiclone	Enhanced sedation	Postoperative apnea, excessive somnolence
	Muscle Relaxants	Cyclobenzaprine, Tizanidine	Synergistic muscle weakness	Respiratory muscle paralysis
	Other CNS Depressants	Ethanol, Gabapentin Pregabalin	Multimodal CNS depression	Fall risk elevate, postoperative respiratory depression
	GABA analogue	Sodium Oxybate	Severe respiratory synergy	Hypotension, syncope, sudden death
	Antihistamines	Meclizine	Enhanced sedation	Somnolence, respiratory depression
	Anesthetics	Isoflurane, Ketamine	Intraoperative synergy	Hypercapnia, hypoxemia
Pregabalin	CNS Depressants	Opioids (morphine), benzodiazepines (remimazolam), sedatives (oxybates)	Additive CNS depression	Increased risk of respiratory depression, profound sedation, hypotension, coma, or death
	Anticonvulsants	Phenytoin, carbamazepine	Induction of microsomal hydroxylation	Possible reduced serum calcifediol levels​​ (vitamin D metabolite)

There were no known specific antidotes for Baclofen and Pregabalin overdose. In cases of confirmed or suspected drug toxicity, the drug should be discontinued immediately to prevent further toxic accumulation. Airway protection is critical during intervention, particularly when performing gastric lavage, but respiratory stimulants should be avoided. If overdose is confirmed, gastric lavage or emesis may be tried to remove unabsorbed drug and promote gastric emptying. In patients with mild toxicity, supportive therapy with close monitoring of vital signs and neurologic status is recommended. For severe cases, mechanical ventilation may be required. Both drugs are predominantly renally excreted. While forced diuresis may enhance elimination in patients with adequate renal function, hemodialysis should be prioritized in renal impairment. This is supported by their pharmacokinetic profiles: Baclofen has a small molecular weight (213 Da), low apparent Vd, and 30% plasma protein binding; Pregabalin’s manufacturer notes significant dialyzability (≈50% clearance in 4 h) (FDA, 2025). Clinical evidence further validates this approach—a 74-year-old woman with Baclofen-induced coma regained neurological function after hemodialysis (Al Marzouqi and Al Alawi, 2023)^,^ consistent with other reports (Riaz et al., 2020; Jeyaselvasenthilkumar et al., 2019; Mishaal et al., 2020; Pathak et al., 2019; Shah et al., 2020; Varma and Bajpai, 2022; Khazneh et al., 2018; Wolf et al., 2018; Takia et al., 2021; Al Marzouqi and Al Alawi, 2023; Gold et al., 2020).

Adverse reactions to Baclofen and Pregabalin predominantly manifest during treatment initiation or rapid dose escalation, necessitating enhanced therapeutic drug monitoring in early-phase administration. Clinicians are advised to prioritize conservative initial dosing strategies when use Baclofen and Pregabalin, initiating therapy at the lower end of the therapeutic range and titrating gradually unless clinical urgency necessitates rapid escalation (Vlavonou et al., 2014). Postoperative contexts require particular vigilance, as blood-brain barrier vulnerability may amplify CNS depression; this demands rigorous evaluation of concomitant CNS depressants (Table 2) with dosage individualization based on hepatic/renal function. Our systematic analysis of 19 clinical cases corroborates the necessity of these preventive strategies in high-risk populations, aligning with pharmacovigilance protocols.

Clinical pharmacists play a crucial role in the identification and individualized monitoring of ADRs. Upon receiving a consultation request, clinical pharmacists first need to clarify the core objectives of the consultation. Then, they use the system to review medical records comprehensively to fully understand the patient’s condition, focusing on key information related to drug therapy, including basic data such as height and weight, current medical history and the evolution of past diseases, allergy history, and complications. They also integrate the patient’s previous medication history and confirmed adverse drug reaction records. Subsequently, they conduct detailed consultations with the patient or their family members in the ward to directly understand the specifics of drug use and the occurrence of adverse reactions. After the consultation, they communicate thoroughly with other members of the medical team (doctors, nurses) to verify the actual implementation of the current drug treatment plan and further confirm the correlation between drug therapy and clinical manifestations. Finally, they provide consultation opinions from the perspectives of medication safety, efficacy, and risk-benefit balance, offering professional pharmaceutical support for clinical treatment decisions. In this case, the clinical pharmacist confirmed the relevance of baclofen combined with pregabalin and CNS inhibition through pharmacological consultation, pharmacological assessment, and adverse reaction judgment, and recommended that the patient stop taking the medication promptly, which played a positive role in the patient’s favorable outcome. Current literature has already demonstrated that clinical pharmacists assist clinicians in assessing adverse drug reactions in complex medication scenarios. In the case reported by Stuhec et al., a clinical pharmacist found that the combination of solifenacin and trazodone produced a synergistic central anticholinergic load. By reorganizing the medication, the pharmacist successfully reversed delirium by replacing solifenacin with dalfonacin (Štuhec, 2013). In another case report, a clinical pharmacist identified the synergistic CNS toxicity between trimethoprim-sulfamethoxazole and antipsychotic drugs. After replacing trimethoprim-sulfamethoxazole with nitrofurantoin, the patient’s condition improved, and hallucinations did not recur (Stuhec, 2014). These cases all demonstrate that the core competence of a clinical pharmacist lies not only in identifying the risks of individual drugs but also in analyzing the synergistic toxic mechanisms (including pharmacodynamics and pharmacokinetics) when multiple drugs are used together. Through systematic pharmaceutical knowledge, they help balance risks and benefits to assist clinicians in selecting treatment plans.

## Conclusion

The present study, through case study analysis and case review, has clarified that there is a high risk of CNS depression in patients with renal impairment when treated with the combination of Baclofen and Pregabalin. Therefore, initiating therapy at reduced starting doses is strongly recommended for all patients, with particular vigilance in renal impairment cases. Dose adjustments should be guided by CLcr when necessary. In the event of an adverse reaction, immediate discontinuation and appropriate therapeutic measures are critical. Clinical pharmacists can play a pivotal role in both identifying and managing adverse drug reactions.​

### Limitation

While this study incorporates a narrative review of pharmacological interactions, we acknowledge that the synthesis of existing evidence does not follow systematic review methodology. However, the use of this approach rather than a systematic review is simply to place the novelty of our case findings in the context of broader clinical observations and to support them with previous cases. This limitation does not diminish the clinical urgency revealed by our case, but rather emphasizes the need for methodological rigor in subsequent investigations.

## Data Availability

The original contributions presented in the study are included in the article/supplementary material, further inquiries can be directed to the corresponding author.
